# The Pardon of Dr. Beale

**Published:** 1856-01

**Authors:** 


### THE PARDON OF DR. BEALE.

It will be remembered by the readers of this Journal that we noticed the extraordinary and untenable grounds of his conviction, and the great injustice of his imprisonment. We were in Philadelphia shortly after the act was said to have transpired for which he was arraigned and finally convicted. From the testimony, we were convinced of the unjustness of the charge, in which opinion we were not alone.

It appears, however, that his friends who believed his incarceration *an outrage* upon the name of justice, persisted in their efforts for his liberation from prison, and were at last fortunately successful. The scene, described as having occurred upon his unlooked-for return to his family, was said to have been most affecting. In the bosom of his family we hope he will find solace and comfort for his crushed feelings and hopes, and although the almost entire profession and the world believe him innocent, still *there* is the opprobium and the stain, which will cling to him through life. That jury, we do not hesitate to say, and we would that we could reach every man who sat upon it, perpetrated an act, when they found him guilty upon such testimony, *worse than murder itself.*

But upon examining the reasons of Governor Pollock for pardoning him, we could not withhold the smile. He recites among the reasons for his liberation, first, that the ends of justice were satisfied by his confinement up to that time,—that he had a dependent family and aged parents to support, and last, (and *least* of all, we presume,) that from the report of the testimony and the opinions of scientific men *he believed he was not guilty!* It he was convinced of his innocence, in the sacred name of justice, why did he allow him to be incarcerated a single hour? How was "justice satisfied by his imprisonment," if he was innocent? The claims of dependent families might be adduced in favor of hundreds of other cases, who are justly suffering the penalty of the law which they have outraged. But in the opinion of the Governor, he outraged no law, and yet he would liberate him *because* "justice was satisfied in its demands, and because too of the size of his dependent family."

His Excellency ought to blush for such a medley of reasoning, for such puerile excuses for a just act of clemency, for his inhumanity in permitting him to remain *one moment* in prison in the face of such an array of public and professional opinion; from which, and the want of testimony to convict him, he himself believed him innocent.

The sole testimony upon which he was convicted was that of the female herself, who may have sworn correctly as to her impressions. Her conduct subsequently, however, to say the least of it, and to give it the most liberal construction, was truly strange and inconsistent with the circumstances of the case. But admit that she spoke truthfully as to her sensations, her testimony should not have been received without the strongest confirmatory proof from other sources. What was produced from others was by no means of that character. As to her fitness to testify to the facts, experience proves that she was not in a condition to do so. Carpenter, whose authority no medical man will doubt on this subject, says, when treating upon the subject of perceptive and intuitional consciousness, "if we fall asleep whilst suffering from bodily pain, we may lose all perception of the cause of that pain, as having its seat in our bodily fabric, and yet remain conscious of a perturbed state of feeling; and when a surgical operation is performed in a state of incomplete anæsthesia, it is obvious that pain is felt without any distinct consciousness of its source, and the patient may subsequently describe his state as an uneasy dream."

What court of justice would commit so silly an act as to admit the testimony of an individual, even partially asleep, as to the probable guilt of an individual, when the external world had no power of making its impression upon the consciousness, so as truthfully to be perceived. Such an act would be taken as proof of its total unfitness for the discharge of its functions, and the jury who would decide upon such testimony, would justly forfeit the respect of all men of sense and judgment.

